# A survey of Autism knowledge and attitudes among the healthcare professionals in Lahore, Pakistan

**DOI:** 10.1186/1471-2431-11-107

**Published:** 2011-11-22

**Authors:** Nazish Imran, Mansoor R Chaudry, Muhammad W Azeem, Muhammad R Bhatti, Zaidan I Choudhary, Mohsin A Cheema

**Affiliations:** 1 Child & Family Psychiatry Department, King Edward Medical University/Mayo Hospital, Lahore, Pakistan; 2 Academic Department of Psychiatry & Behavioural Sciences, King Edward Medical University/Mayo Hospital, Lahore, Pakistan; 3 Riverview Hospital for Children and Youth, Department of Children and Families CT, Yale Child Study Center, Middletown, CT, USA; 4 Department of Psychiatry& Behavioural Sciences, King Edward Medical University/Mayo Hospital, Lahore, Pakistan; 5 Academic Department of Psychiatry & Behavioural Sciences, King Edward Medical University/Mayo Hospital, Lahore, Pakistan; 6 Department of Physiology and Cell biology, University of Health Sciences, Lahore, Pakistan

## Abstract

**Background:**

The diagnosis and treatment of Autism in Pakistan occurs in multiple settings and is provided by variety of health professionals. Unfortunately, knowledge and awareness about Autism is low among Pakistani healthcare professionals & the presence of inaccurate and outdated beliefs regarding this disorder may compromise early detection and timely referral for interventions. The study assessed the baseline knowledge and misconceptions regarding autism among healthcare professionals in Pakistan which can impact future awareness campaigns.

**Methods:**

Physicians (psychiatrists, pediatricians, neurologists and family physicians) and non-physicians (psychologists and speech therapists) participated in this study. Knowledge of DSM-IV TR criteria for Autistic Disorder, beliefs about social, emotional, cognitive, treatment and prognosis of the disorder were assessed. Demographic information regarding the participants of the survey was also gathered.

**Results:**

Two hundred and forty seven respondents (154 Physicians & 93 Non-physicians) participated in the study. Mean age of respondents was 33.2 years (S.D 11.63) with 53% being females. Reasonably accurate familiarity with the DSM IV-TR diagnostic criteria of Autistic Disorder was observed. However, within the professional groups, differences were found regarding the utilization of the DSM-IV-TR criteria when diagnosing Autistic Disorder. Non-Physicians were comparatively more likely to correctly identify diagnostic features of autism compared with Physicians (P-value <0.001). Significant misunderstandings of some of the salient features of autism were present in both professional groups.

**Conclusion:**

Results suggests that current professionals in the field have an unbalanced understanding of autism due to presence of several misconceptions regarding many of the salient features of autism including developmental, cognitive and emotional features. The study has clinical implications and calls for continued education for healthcare professionals across disciplines with regards to Autism in Pakistan.

## Background

Autism Spectrum Disorders (ASD) are pervasive and lifelong neurodevelopmental disorders characterized by impaired socialization, impaired verbal and nonverbal communication, and restricted interests and repetitive patterns of behavior [1]. It is believed to be one of the fastest growing disabilities in children [2,3]. Although knowledge and research on ASDs are on the rise worldwide [4], most studies across different nations, have reported wide variation among healthcare professionals regarding diagnosis, treatment and prognosis of Autism. An earlier survey by Stone et al. (1987) clearly demonstrated that many professionals in various disciplines did not possess accurate knowledge about Autism and its manifestations in children and adolescents [5]. Autism was thought to be a temporary disability and rooted in emotional factors. Variations were also found between the primary care providers (family physicians, pediatricians & neurologists) and specialists (child psychiatrists, speech therapists and psychologists) in a survey of autism knowledge in the United States[6]. Health care workers (HCW) in Sub-Saharan African subcultures were also found to have various misconceptions regarding etiology, treatment and prognosis of Autism spectrum disorders [7,8]. A survey conducted by the African Network for the Prevention and Protection against Child Abuse and Neglect(ANPPCAN) in 2007 showed low to moderate level of knowledge about autism among the various categories of healthcare workers(HCW) with highest level of awareness in healthcare workers of psychiatric facilities in the region [9]. Looking at the studies from subcontinent, Indian psychiatrists, psychologists, and pediatricians generally agreed on characteristics required to diagnose Autism [10], while in another study done in Karachi, Pakistan, General Practitioners who were less than 30 years of age and five or fewer years after acquisition of their medical degrees, were found to be more informed and accurate in their understanding of Autism[11].

In Pakistan, child psychiatric services are still in their infancy. There are no formal referral pathways to psychiatric centers for children. The diagnosis and treatment of Autism occurs in multiple settings and is provided by variety of health professionals including family physicians, pediatricians, neurologists, psychiatrists, psychologists and speech & language therapists. Most of these health professionals have little or no formal training in child and adolescent psychiatry [12,13]. Thus many questions regarding this puzzling disorder remain unanswered in Pakistan. Unfamiliarity with DSM-IV TR diagnostic criteria of Autistic Disorder and the presence of inaccurate and outdated beliefs held by Pakistani healthcare professionals may have delayed the positive effects which early interventions have been shown to have upon the prognosis [14,15]. To date, little research has been published from developing countries regarding the above mentioned issues. The present study seeks to address this knowledge gap in Pakistan.

## Methods

The Institutional Review Board of King Edward Medical University, Lahore, Pakistan approved this study. The study was conducted between August 2009 and January 2010 in Lahore, which is the second largest city of Pakistan. Respondents were recruited in two ways. All psychiatrists, psychologists, neurologists, pediatricians, and speech & language therapists with minimum of six months of clinical experience in all five teaching hospitals within the public sector in Lahore were invited to participate in the study. In addition, family physicians were recruited by distributing the questionnaire in two consecutive Continuing Medical Education meetings organized by Pakistan Academy of Family Physicians (Lahore Chapter). The speech therapists who participated were also recruited from an Institute in Lahore, Pakistan which at the time of study was the only one providing a qualification course in speech therapy in the city.

The survey form used, comprised of four sections (Additional file 1). The first section queried participants about their background and experience with autism. The second listed ten behaviors or characteristics of autism according to DSM IV-TR diagnostic criteria. Respondents were asked to rate the characteristics as "Necessary", "Helpful but Not Necessary" or "Not Helpful" for the diagnosis of Autistic Disorder. Among the items of each of the three options, only the category of "Necessary" comprised factors representing the correct diagnosis of Autism. The three categories were mutually exclusive. When multiple categories were assigned to the same item, those responses were discounted.

The third segment was a modified version of a section of Autism Survey as developed by Stone et al. [5] which has been shown to demonstrate rigorous psychometric properties [16]. Twenty-two beliefs regarding social, emotional, cognitive, treatment and prognostic aspects of Autism were presented. Some items of the standardized Autism Survey were substituted with more common mistaken notions regarding Autism in Pakistan. These were established while piloting the four section questionnaire. The respondents were instructed to rate each statement either as "Agree", or "Not Sure", or "Disagree".

The final part of the survey queried various treatment options considered helpful by the professionals.

The data was analyzed by using SPSS Version 17.0 [17]. Descriptive statistics of socio-demographic information were gathered. For the purpose of analysis, healthcare professionals were separated into two groups; Physicians (family physicians, pediatricians, psychiatrists, neurologists) and non-physicians (psychologists, speech and language therapists). For all purposes, a p-value of <0.05 was considered statistically significant.

## Results

The total number of surveys distributed was three hundred and twenty-five. Two hundred and sixty three were returned representing a response rate of 80.9%. Sixteen questionnaires were incomplete and excluded. The final total of respondents numbered two hundred and forty seven. Mean age of respondents was 33.2 years (S.D 11.63) with 53% being females. Physicians were hundred and fifty four (48 family physicians, 63 pediatricians, 36 psychiatrists and 7 neurologists). Non-physicians were ninety three (79 psychologists and 14 speech therapists). A significantly higher number of physicians than non-physicians had encountered youths with Autism in their clinical practice, i.e. 77 compared with 58 (P value = 0.042). About 70% of both groups were practicing for less than five years. An increase in the number of years of practice was directly related to a larger number of affected youths seen (P value <.001).

Table 1 compares the rankings of the relevance of various aspects/behaviors when considering a diagnosis of Autistic Disorder as considered by Pakistani physician and non-physician groups.

**Table 1:** Comparative Percent Rankings of Characterstics as "Necessary" For Diagnosis of Autism Among Physicians and Non Physicians Group.

Rank	PHYSICIANS	Percentage	Rank	NON -PHYSICIANS	Percentage
	***Diagnostic characteristics***			***Diagnostic characteristics***	

1	Social interaction difficulties	73.2	1	Social interaction difficulties	95.7

2	Lack of social responsiveness	71.1	2	Lack of social responsiveness	92.4

3	Lack of eye contact	68.0	3	Rigid or stereotyped play activities	90.2

4	Language delays	65.4	4	Lack of eye contact	88.2

5	Rigid or stereotyped play activities	59.7	5	Need for sameness, resistance to change in routines	85.7

6	Onset of symptoms before 36 months	56.2	6	Language delays	78.7

7	Need for sameness, resistance to change in routines	53.6	7	Preoccupation with objects.	72.7

8	Unusual mannerisms such as finger flicking	47.6	8	Peculiar speech characteristics	69.2

9	Peculiar speech characteristics	43.5	9	Onset of symptoms before 36 months	59.1

10	Preoccupation with objects.	41.1	10	Unusual mannerisms such as finger flicking	54.9

Table 2 shows the responses of Pakistani physicians and non-physician groups regarding general beliefs about Autism, its prognosis and treatment.

**Table 2:** Comparison Of Physicians and Non- Physicians Regarding General Beliefs about Autism.

Sr #	Beliefs	Physicians N = 154 Positive Responses (%)	Non -Physicians N = 93 Positive Responses (%)	P -Value
1	Autism can occur in mild as well as extreme form	84.8	93.4	.004*

2	Children with autism usually grow up to be schizophrenic adults	24	17.4	0.306

3	Autism is an emotional disorder	40.4	23.9	.001*

4	Most children with autism are also mentally retarded	34.9	46.7	0.17

5	It is difficult to distinguish between autism and childhood schizophrenia	37.9	28.6	0.077

6	Autism occurs more commonly among higher socio economic and educational levels	25.7	20.7	0.667

7	Autistic children's withdrawal is mostly due to cold, rejecting parents	42.5	35.6	0.424

8	Most children with autism have special talents or abilities	55.9	69.2	0.061

9	Autism is a rare condition in this country as compared with the West	33.6	36.4	0.852

10	Autism is under-recognized and often missed in general practice	85.5	70.1	.003*

11	There is a lack of awareness regarding autism among professionals in Pakistan	86.7	76.1	0.072

12	Autism is a communication disorder	56	46.7	.022*

13	Children with autism do not show social attachments, even to parents	57.8	74.2	.009*

14	It is impossible to tell if a child has autism before four years of age	23.1	27.2	.001*

15	Autism exists only in childhood	43.1	50.5	.028*

16	Even with early intervention, the prognosis for independent community functioning of individuals with autism is poor	32.9	46.7	.028*

17	Autism is a developmental disorder	51	71	.009*

18	With the proper treatment, most children with autism eventually "outgrow" autism.	49.3	44	0.469

19	Autism is a lifelong condition	44.6	51.6	0.561

20	Children with autism are "untestable."	34	20.2	.003*

21	Parental counseling on training techniques is one effective treatment of autism.	76.2	96.8	.001*

22	Dietary intervention is one of treatment options	35.6	50	0.038

(% given are the respondents answering in affirmative)

Regarding interventions, psychotropic medications were considered helpful by 70% of respondents. 27.1% regarded mood stabilizers as useful; psychostimulants were endorsed by 24.8%, followed by antipsychotics, 23.1%; antidepressants, 18.3% and hypnotics, 4.2%. Speech therapy and special educational interventions were found helpful by 75% and 68% of respondents respectively.

## Discussion

Based upon our review of the relevant medical literature, the current study is the first to examine the knowledge of standardized diagnostic criteria of Autistic Disorder and the beliefs about the disorder held by Pakistani healthcare professionals from various disciplines. In general, the different professionals appear to have a similar impression of the disorder as represented by the social deficits, communication difficulties and restricted interests demonstrated by children with Autism. An important finding is that the early onset of the disorder, that is before 36 months of age; (a diagnostic hallmark of Autism) is either unknown/undervalued and/or not applied in the diagnostic process. Studies have found parents of children with Autism noticing and then voicing their concerns to health professionals, when their children were between the ages of fifteen to nineteen months [18,19]. However this recognition does not necessarily translate to an early diagnosis by the healthcare professionals. One of the main presentations of Autism is speech delay but that is not considered atypical in Pakistan at thirty six months or beyond, resulting in many children being assessed, referred and diagnosed far beyond early childhood. Accounting for this lack of emphasis on early age of presentation may be the lack of understanding of typical presenting complaints, reluctance and fear of labeling a child at an early age, and/or the overlap of symptoms of autism with other comorbid disorders such as cognitive delay. Changing the negative opinions and beliefs of HCW regarding autism should encourage appropriate help seeking behaviors among parents leading to encouragement of early interventions which are essential for favorable prognosis.

Differences between the groups were shown by discrepant views about the relevance of particular characteristics used when diagnosing Autism. The discrepancy in knowledge found among healthcare workers was not totally unexpected due to health service structure in Pakistan. This is also consistent with the results of previous studies documenting some differences and misperceptions about various aspects of autism across disciplines [5,6,11,20,21]. Since child specialty centers in Pakistan are few, many of the children with Autism and intellectual disabilities are first referred to special education centers by various professionals, usually without comprehensive assessments and standardized diagnostic evaluations. Youth in the Autism spectrum are more likely to be brought to psychiatric facilities to treat externalizing behaviors and aggression, intellectual delays affecting motor, feeding, or late onset of developmentally appropriate self-care functions or epilepsy. These difficulties are considered to be areas of practice of psychiatrists and psychologists in Pakistan. Family physicians and pediatricians in most cases have no formal child psychiatry exposure during their training and feel less confident in assessing and managing these children. Similar to the global trend, in Pakistan, family physicians and pediatricians are typically the first medical professionals to whom parents will express their concerns regarding the child's development. The need for these medical practitioners to be equipped with more knowledge about Autism cannot be over emphasized in order to aid early diagnosis and interventions[22,23]. On the other hand majority of psychologists and speech therapists have internships in special education centers during their training and therefore perhaps are more aware of the typical presentations and diagnostic criteria of Autism. Our results differ in this respect to a study done in Nigeria in which undergraduate medical students were more likely to recognize symptoms and signs of autism compared to nursing and Psychology students [24].

Respondents in our study also shared several misconceptions regarding autism across social, emotional, cognitive and general descriptive features of autism, which are consistent with previous studies showing significant differences in healthcare professionals, parents, teachers and medical students' knowledge regarding possible causes, cognitive profiles, treatment and prognosis of autism[5,24-26].

DSM IV-TR diagnostic criteria require delays and impairments being present in the first 36 months of life and respondents in this study were likely to agree that Autism is a developmental disorder, but also classified it as an emotional disorder. The mistaken overlap of these pathophysiologic distinct categories may be linked to the relative low incidence of recognition of impairments in early life as one of the features for diagnosing Autism. Non Physicians were again more likely to consider autism as a developmental disorder. The participating health professionals were also likely to endorse the outdated view that the cause of social withdrawal in Autism was a parental bonding and child attachment difficulty. Even though the etiology credited once to aloof, rejecting parenting, now this has been shown irrelevant by researchers in the field [27].

Outdated views regarding the course of Autism were also observed with the majority believing that Autism exists only in childhood and many children will outgrow the disorder with proper treatment. This is similar to results reported by previous studies [5].

While not corroborative with the previously noted survey misconception, study respondents endorsed that a child with Autism is more likely to grow up to be an adult with Schizophrenia, consistent with speculation of Autism as an early form of Schizophrenia [27]. This finding may support that respondents have difficulty distinguishing between Autism and Childhood-Onset Schizophrenia. This response further supports that healthcare professionals in Pakistan feel less comfortable in diagnosing children with Autism. Recent research on the other hand shows less likelihood of the presence of autism symptoms in patients with schizophrenia [28].

Overall, the results of our study have significant clinical implications. The fact that more than a quarter of study participants were not likely to endorse the need for speech therapy and special education services, they were also less likely to advocate for these much needed services for children with Autism in Pakistan.

In order to improve child mental health in Pakistan, physicians and other relevant professionals working in large cities do have a significant role to play in disseminating accurate information and providing training to other healthcare workers about Autism. The need to improve the areas of knowledge gaps identified by this study cannot be overemphasized. Some of the ways to help with this huge task may be to incorporate important aspects of child developmental disorders in undergraduate and post graduate medical curriculum. The visits to special schools especially for pediatricians and family physicians during their training could be beneficial. Various conference forums and the effective use of media to promote evidence based medicine and the erosion of myths about Autism held by the general population and in particular among the healthcare professionals is much needed in Pakistan [29-31]. More emphasis is needed to implement policies to change the negative attitudes of health professionals regarding various aspects of autism to promote care for children with Autism Spectrum Disorders in this region. It should include continuous medical education of Pediatric and Psychiatric nursing staff as well because they are expected to provide counseling to families of these children.

Several limitations exist with the design of the current study and must be considered when interpreting and placing value on the results obtained. The largest limitation was the sample size. Although several efforts were made to increase the sample size by visits to all teaching hospitals in public sector in Lahore as well as approaching health care professionals at various conferences, the resulting number of participants was still somewhat low. It is possible that the results do not reflect the perspectives of the professionals as a whole.

Also the survey developed for the present study was based on several examples from the literature, but the survey itself was not validated. There is the possibility that questions were confusing despite the fact that the study questionnaire was piloted among a group of healthcare professionals and the identified deficiencies eliminated. Generalization to other settings is another limitation.

Regarding a weakness in study design, (since it has been shown that persons generally answer in the affirmative when not sure about a particular item), asking respondents to list behaviors or characteristics they use in diagnosing autism rather than presenting a list upon which to comment, may have led to a more accurate knowledge of diagnostic criteria of Autism, rather than a possible overestimate.

Another difficulty encountered was the absence of an expert group in the study, included in previous studies [5,10,32], introducing a weakness for comparison. Although there are many highly qualified professionals in Pakistan, without dedicated Autism centers and standard criteria for determining health professionals' expertise in Autism, it was difficult to establish such a group for comparison in this study.

## Conclusion

Despite the limitations of the study, the results provide an important overview of the diagnostic practices and knowledge of Autism from a low income developing country with absence of child mental health policy. Results suggests that current professionals in the field have an unbalanced understanding of autism due to presence of several misconceptions regarding many of the salient features of autism including developmental, cognitive and emotional features. The study has clinical implications and calls for continued education for healthcare professionals across disciplines with regards to Autism in Pakistan.

Future studies are needed which should include multiple sites across Pakistan encompassing both urban and rural settings with a larger sample size. These studies can provide baseline data to guide policies and planning of healthcare delivery to children with Autism and other developmental disorders in Pakistan.

## List of Abbreviations Used

(ASD): Autism Spectrum Disorder; (DSM- IV): Diagnostic and Statistical Manual of Mental Disorders, 4^th ^edition.; (HCW): Health care Workers.

## Competing interests

The authors declare that they have no competing interests.

## Authors' contributions

**NI: **Conception & design, data analysis and interpretation, article drafting. **MRC**: conception & design, data collection, analysis. M**WA**: Design of the study, manuscript writing, critical revision. **MRB**: Study plan, write up and critical revision. **ZIC**: literature search, data collection & interpretation. **MAC**: Design, data analysis, manuscript writing.

All the authors read and approved the final draft of the article.

## Pre-publication history

The pre-publication history for this paper can be accessed here:

http://www.biomedcentral.com/1471-2431/11/107/prepub

## Supplementary Material

Additional file 1 **The Autism Survey Questionnaire**. The survey form used in the study. Click here for file
