# Supplementary material for: A survey of Autism knowledge and attitudes among the healthcare professionals in Lahore, Pakistan
Source: BMC Pediatr. 2011 Nov 22;11:107. doi: 10.1186/1471-2431-11-107 (PMC3250946; doi:10.1186/1471-2431-11-107)
Supplement: Additional file 1 — The Autism Survey Questionnaire. The survey form used in the study. [file 1471-2431-11-107-S1.DOC]

Dear Respected Sir/Madam,

We are undertaking a research on Health Professional’s knowledge and beliefs about Autism. Kindly fill in the accompanied Questionnaire. Answering the Questions also means giving informed consent about taking part in this study. **For the purpose of anonymity, kindly do not write your name.**

Thank you for your kind cooperation.

Research Team.

**Questionnaire**

**Age:** ______________ **Gender:**  Male / Female

**Qualifications: _____________(MBBS, MSc, MS, FCPS, MRCP, Diplomate American Board, Dip Paeds, Dip speech etc)**

**Area of speciality:**  ___________ *( Psychiatrist, Pediatrician, Gen. Practitioner etc.)*

**Duration of clinical experience:** 0-1 year 1-5 years more than 5 years

**In your career, have you encountered any cases of Autism?**  Yes No

**For the following, check one box for each behaviour or characteristic, you think are 1- Necessary,**

**2- Helpful but not necessary and 3- Not helpful for autism diagnosis.**

**Behaviours/Characteristics Necessary Helpful but not Not Helpful**

**necessary**

1. Language Delays -----------------------------------------------------------------------------------------
2. Lack of eye contact ------------------------------------------------------------------------------------
3. Need for sameness, resistance to change in routine -------------------------------------------------
4. Peculiar speech characteristics -----------------------------------------------------------------------
5. Lack of social responsiveness ---------------------------------------------------------------------------
6. Rigid or stereotyped play activities ----------------------------------------------------------------------
7. Onset of symptoms before 36 months -----------------------------------------------------------------
8. Unusual mannerisms such as finger flicking ------------------------------------------------------
9. Preoccupation with objects ---------------------------------------------------------------------------
10. Social interaction difficulties -------------------------------------------------------------------------

**For those diagnosed with autism, what interventions you think are helpful.**

**Check all the boxes that apply.**

Medication: Antipsychotics Antidepressant Mood stabilizers

Psychostimulants Hypnotics Others /*Please specify________________*

Speech Therapy

Special Education

Referral to Psychiatrist

Referral to Psychologist

Others Please specify____________________________________________________ **_____________**

**For each of the following statements, check the box best represents your views.**

**Disagree**

**Agree**

**Not Sure**

1. Autism can occur in mild as well as extreme forms.-------------------------------
2. Autistic children usually grow up to be schizophrenic adults---------------------
3. Autism is an emotional disorder.------------------------------------------------------
4. Most Autistic children are also mentally retarded----------------------------------
5. It is difficult to distinguish between Autism and childhood schizophrenia.---
6. Autism occurs more commonly among higher socioeconomic and

educational levels.---------------------------------------------------------------------

1. Autistic children’s withdrawal is mostly due to cold, rejecting parents---------
2. Most autistic children have special talents or abilities.----------------------------
3. Autism is a rare condition in this country as compared with the West.---------
4. Autism is under-recognized and often missed in general practice.---------------
5. There is a lack of awareness regarding Autism among professionals in

Pakistan.---------------------------------------------------------------------------------

1. Autism is a communication disorder. ------------------------------------------------
2. Autistic children do not show social attachments, even to parents---------------
3. It is impossible to tell if a child is Autistic before 4 years of age.----------------
4. Autism exists only in childhood.------------------------------------------------------
5. Even with early intervention, the prognosis for independent community

functioning of Autistic individuals is poor.----------------------------------------

1. Autism is a developmental disorder.-------------------------------------------------
2. With the proper treatment, most autistic children eventually “outgrow”autism.
3. Autism is a lifelong Condition.-------------------------------------------------------
4. Autistic children are “untestable.”---------------------------------------------------
5. Parental counseling on training techniques is one effective treatment of

Autism.----------------------------------------------------------------------------------

1. Dietary intervention is one of treatment options.-----------------------------------

**Are you aware of any specialized centre for autism in Pakistan especially in Lahore, if yes please give details:**

**___________________________________________________________________________________**

**___________________________________________________________________________________**

**Where would you refer a child if you diagnose or suspect autism?**

**__________________________________________________________________________________________**

**__________________________________________________________________________________________**

**What other comments would you like to add about your experiences with Autism or related disorders?**

**____________________________________________________________________________________**

Thank you for your help. If you will like to be informed about a training workshop on Autism, you may give your contact number/e-mail on this form.

Contact / E-mail: ____________________________________________________________________________
